# Automatic segmentation of retinal layers in OCT images with intermediate age-related macular degeneration using U-Net and DexiNed

**DOI:** 10.1371/journal.pone.0251591

**Published:** 2021-05-14

**Authors:** Jefferson Alves Sousa, Anselmo Paiva, Aristófanes Silva, João Dallyson Almeida, Geraldo Braz Junior, João Otávio Diniz, Weslley Kelson Figueredo, Marcelo Gattass

**Affiliations:** 1 Federal University of Maranhão, São Luís, Maranhão, Brazil; 2 Pontifical Catholic University of Rio de Janeiro, Rio de Janeiro, Rio de Janeiro, Brazil; University of Florida, UNITED STATES

## Abstract

Age-related macular degeneration (AMD) is an eye disease that can cause visual impairment and affects the elderly over 50 years of age. AMD is characterized by the presence of drusen, which causes changes in the physiological structure of the retinal pigment epithelium (RPE) and the boundaries of the Bruch’s membrane layer (BM). Optical coherence tomography is one of the main exams for the detection and monitoring of AMD, which seeks changes through the evaluation of successive sectional cuts in the search for morphological changes caused by drusen. The use of CAD (Computer-Aided Detection) systems has contributed to increasing the chances of correct detection, assisting specialists in diagnosing and monitoring disease. Thus, the objective of this work is to present a method for the segmentation of the inner limiting membrane (ILM), retinal pigment epithelium, and Bruch’s membrane in OCT images of healthy and Intermediate AMD patients. The method uses two deep neural networks, U-Net and DexiNed to perform the segmentation. The results were promising, reaching an average absolute error of 0.49 pixel for ILM, 0.57 for RPE, and 0.66 for BM.

## 1 Introduction

According to data from World Health Organization [1], it is estimated that at least 2.2 billion people have visual impairment or blindness, of which at least 1 billion have some visual impairment that could have been avoided or has not yet been addressed. The five leading causes of blindness are: uncorrected refractive errors, cataracts, age-related macular degeneration, glaucoma, and diabetic retinopathy.

Age-related macular degeneration (AMD) is an eye disease that affects people over 50, being one of the main causes of visual impairment in elderly patients [2]. It is a progressive and degenerative condition, resulting from aging, related to changes in the structure of the retina, causing symptoms of loss of central vision in one or both eyes due to photoreceptor dysfunction or loss [3]. It can be presented in two forms, the most common being dry or non-exudative, identified in 85 to 90% of cases. This form is characterized by the presence of drusen and/or pigmentation alteration and causing slower visual loss. The neovascular or exudative form occurs in 10 to 15% of cases, however, it is responsible for 90% of blindness due to AMD [3]. Druses are extracellular materials accumulated between the retinal pigment epithelium (RPE) layer and the Bruch’s membrane layer (BM) layer. The presence of a certain number of drusen is normal with advanced age. However, the abundant presence of drusen is a common initial indicator of AMD.

The U.S National Library of Medicine (NLM) estimates that AMD affects about 170 million people worldwide. The prevalence rate is expected to increase in the coming decades, as the proportion of elderly people in the population increases. The estimate for 2040 is that 288 million people will be affected by the disease [4].

The clinical diagnosis of AMD is commonly made through retinography (fundus photograph), however, optical coherence tomography (OCT) has been widely used in conjunction with retinography.

OCT has emerged as a viable option for diagnosing retinal changes [5]. It is a non-invasive imaging technology that allows the visualization of biological tissues in vivo in sectional sections with high resolution. Thus, it soon gained great importance in the diagnosis of macular diseases such as AMD. Druses, neovascularizations, fibrovascular scars, and intraretinal or subretinal macular fluid are typical changes in the retina that are detectable in optical coherence tomography.

The optical coherence tomography image allows the evaluation of changes in the retinal pigment epithelium (RPE) and, hence, of drusen. Compared to the other recommended tests, it was evidenced that the OCT is more sensitive to the detection of pathological changes related to AMD. It was found that 57% of the evaluated eyes showed changes perceived only through OCT and not seen through other exams, such as angiofluoresceinography [6]. Another advantage of retinography is the rapid acquisition, eliminating the use of contrasts and thus the risks of allergic reactions.

Early diagnosis of AMD is helpful for monitoring the disease progression and for complications including the onset of wet AMD. When this diagnosis is made from the OCT, it takes place with the visual analysis of the various images generated, and if we take into account that a specialist attends and accompanies several patients per day, technological support for clinical practice is necessary to minimize evaluation errors. Therefore, in this work, we focus on the segmentation of the limits of the ILM, RPE, and Bruch’s membrane of OCT B-scan images of AMD and normal patients. There are two reasons for the focus on these layers. The first reason is that, as the ILM layer is the upper limit of the retina and the BM layer is the innermost limit of the retina, the physiological aspects and morphological structure of the retina can be altered by AMD being detected and visualized in these layers. The second reason is that drusen are formed between RPE and BM, so the boundaries of the RPE and BM layers can indicate data such as size, shape, location, and quantity of drusen. Therefore, we verify the importance of segmentation of these layers to support the detection and diagnosis of AMD.

Given this scenario, this article proposes an automatic method of segmentation of the ILM, RPE, and BM layers of the retina in OCT B-scans images. The proposed method is carried out in four stages: (a) pre-processing (using the bilateral filter); (b) definition of the area of interest (using various image processing techniques); (c) initial segmentation (based on U-net, deep neural network (CNN) model); (d) final segmentation (based on DexiNed, deep neural network architecture for edge detection). Among the contributions of this work, we highlight the proposal of a robust methodology capable of segmenting the layers of the retina in a fully automated manner.

This article is organized into 5 sections. Section 2 describes the proposed method. Section 3 presents the results, Section 4 discusses the results. Finally, Section 5 presents a conclusion about the method developed.

### 1.1 Related works

Segmentation of the retina’s boundary layers in OCT images is a challenging task since it is a complex image that presents a large amount of noise and deformations caused by pathologies (drusen, drusenoid PED, geographic atrophy) that change the characteristics of the edges of the layers. Several studies have been published demonstrating methodologies for segmenting the edges of retinal layers.

In [7] a method is presented for segmentation of the total retina, which lies between the internal limiting membrane (ILM) and the retinal pigment epithelium (RPE), and the RPEDC, which lies between the RPE and the Bruch’s membrane. To validate the method, 220 B-scans extracted from 20 volumes are used. All patients had age-related macular degeneration (AMD) in the intermediate stage and with druses, and some with geographic atrophy. The method is based on graph cutting. As a pre-processing step, noise reduction is performed through a rectangular mean filter. From the resulting image, a graph is constructed with the pixel values as weights. The search for each edge is performed sequentially. Considering all layers, the algorithm showed an average difference of 0.95 pixels, a value even smaller than the difference in segmentation between two specialists. Together with the one developed by [8], these works refer to the development of the DOCTRAP software (Duke OCT Retinal Analysis Program) designed for use in OCT segmentation research.

In [9], a framework is presented that combines convolutional neural networks (CNN) with graph search algorithms to segment nine retinal edges in OCT images. The method was validated with 60 volumes (2915 B-scans) of twenty eyes from people with non-exudative AMD. The CNN is trained with the characteristics of the layers’ edges to estimate the positioning of the edges of the eight layers. Then, these values are put through a graph search algorithm for the final definition of the boundaries. The results found were compared to the results obtained by the DOCTRAP and OCTExplorer segmentation software. These results were superior to those obtained by the OCTExplorer software but still inferior to those obtained by the DOCTRAP software.

[10] presents a method for segmenting the edges of retinal layers in OCT images. Three public databases ([7, 11, 12]) were used, two with a total of 210 B-scans of patients without a pathology and the other with 220 B-scans of patients with intermediate AMD. Two models based on the combination of Convolutional Neural Network (CNN) and Long Short Term Memory (LSTM) are created, 1 to segment eight retina layers and another to segment three layers. CNN is used to extract a region of interest and detect the layers’ edges, while LSTM is used to track the layer boundary. This model is trained with a mix of normal and AMD cases. The results show an average absolute error (EMA) in pixels of 1.30±0.48 *μm*, less than the error of marking the bases of 1.79 ± 0.76 *μm*.

In [13], a new linearly parameterized conditional random field model (LP-CRF) is proposed to segment the retinal layers’ edges OCT images. Two public databases ([7, 11]) were used, one with 107 B-scans from patients without pathology and the other with 220 B-scans from patients with intermediate AMD. The proposed LP-CRF comprises two convolution layers to capture each region’s appearance and layer boundary, the relative weights of the previous form, and an additional term based on the similarity of appearance of the adjacent boundary points. All types of energy are learned in conjunction with a Support Vector Machine. The proposed method segments all the retina’s limits in a single step, and for the images without pathology, eight edges are extracted and with AMD three edges. The mean absolute error reached was 1.52±0.29 *μm* pixels for the segmentation of 8 boundaries in the Normal data set and 1.9±0.65 *μm* pixels for three edges in the combined AMD and Normal data set.

[14] proposed a method for segmenting the edges of retinal layers in OCT images. They used two image databases, one for pediatric patients without a history of pathologies and intermediate AMD patients and pathologies. The method used a trained recurrent neural network (RNN) as a fragment-based classifier to segment seven layers boundaries in the first base and three edges in the second base. The results indicated that the RNN architecture is a viable alternative to CNN for image classification tasks that exhibit a clear sequential structure. Compared to CNN, RNN showed a slightly smaller absolute mean error.

A method for segmenting the edges of the ILM, RPE, and BM layers is presented in [15]. The method was tested on two public databases with normal patients and with intermediate AMD, one provided by [16] with 384 volumes, the other account with 19 volumes made available by [7]. The deep neural network architecture, capsule network, was used to do the initial segmentation of the edges of three layers of the retina, where it classifies fragments extracted from OCT images in the four classes (ILM, RPE, BM, and fundus). To refine the segmentation, the author made use of the graph cut technique, which is applied to the neural network probability maps. As a result, the method reached, respectively, for the two bases, 0.75 and 2.04 average of the mean absolute error for the ILM, 0.93 and 1.97 for the RPE, 1.09 and 2.05 for BM.

In [17], a new method is presented to segment ILM, RPE, and BM in OCT images. Three image bases were used to train and validate the method, with healthy patients’ images and intermediate AMD. The authors use an ensemble method called deepForest, which uses several Random Forests to generate the classification of patches extracted from OCT images. As pre-processing, normalization of pixel intensities is applied. The patches are divided into four classes, internal limiting membrane, retinal pigment epithelium and druse complex (RPEDC), Bruch’s membrane, and fundus. After classification, a graph-based technique developed by the author is applied to refine segmentation. As a result, the method achieved average errors of 0.81, 1.35, and 1.23 pixels for the three bases, respectively.

[18] developed a method for segmentation of retinal layers (ILM, RPE, and BM) in OCT images based on the wave algorithm, a mathematical model of the equation of the potential fluid energy in fluid mechanics. The method uses the base provided by [16] and uses the absolute mean error to evaluate the results. The method obtained an EMA of less than 1.5 pixels in all evaluations.

In the above-mentioned works, we can highlight that everyone uses some graph search techniques to generate the final segmentation of the edges of the retinal layer. The method proposed in this work uses a deep neural network specialized in edge detection to make the final segmentation step.

## 2 Materials and methods

**Fig 1:**
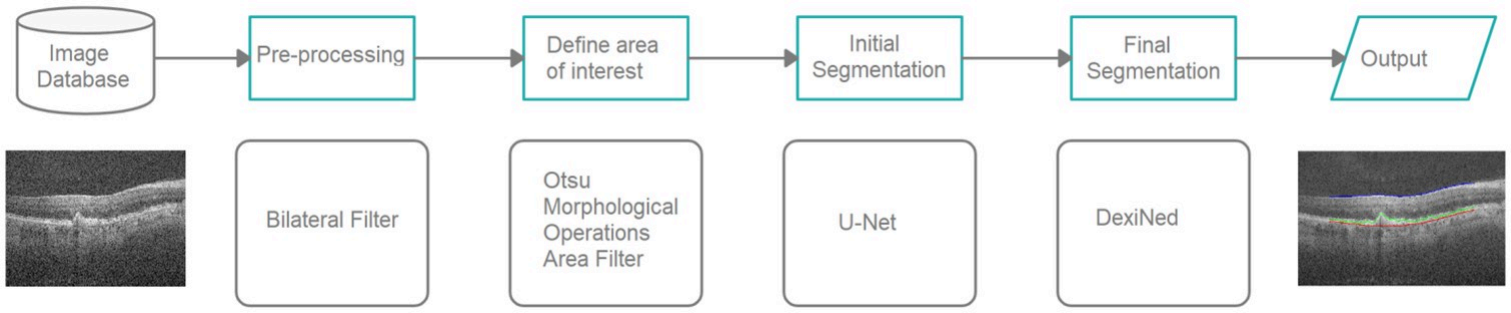
Flowchart of the segmentation method.

The proposed method for segmentation of retinal layers (ILM, RPE, and BM) in OCT images have four steps: pre-processing, the definition of the area of interest, initial segmentation, and final segmentation. Fig 1 shows the method flowchart.

### 2.1 Image dataset

The OCT image base used in [16] is composed of 269 volumes of dry AMD eyes and 115 control volumes (eyes considered normal). The images were acquired in four clinics by SD-OCT scanners manufactured by the company Bioptigen, Inc (Research Triangle Park, NC). The base is composed of individuals who met the following inclusion criteria: between 50 and 85 years of age, exhibiting intermediate AMD with large drusen (≥125 *μm*) in both eyes or large drusen in one eligible eye and advanced AMD in the other eye, without a history of vitreoretinal surgery or ophthalmic disease that may affect the accuracy of any eye. Control subjects were enrolled with the same inclusion criteria for the AREDS2 protocol, except that they should have no evidence of macular druse or AMD in either eye at the initial visit or in the follow-up years. The volumes were acquired in a rectangular region of 6.7 *mm* × 6.7 *mm* centered on the fovea with a rapid scan protocol, resulting in volumes of 1,000 × 512 × 100.

The SD-OCT exams provided also provide the ground truth image of the borders that limit the total retina (between the internal limiting membrane and Bruch’s membrane), as well as delimiting the retinal pigmented epithelium associated with the drusen complex (when existing). The segmentation was initially performed in an automated way by the DOCTRAP software, developed by Duke University, and submitted to review and adjustments by specialists certified by the Duke Advanced Research in Spectral Domain OCT Imaging Laboratory.

After segmentation, the specialists manually marked the central point of the fovea and defined as a region of interest in each volume, a cylinder centered on this point, with a diameter of 5 *mm*.

### 2.2 Pre-processing

To attenuate the noise present in the images of the OCT exam and to highlight the edges, we used the bilateral filter, proposed in [19]. It is a non-linear smoothing filter, which preserves the edges and blurs other regions. To reach this result, each pixel of the image is replaced by a weighted average of the neighboring pixels, considering the geometric proximity and its photometric similarity.

**Fig 2:**
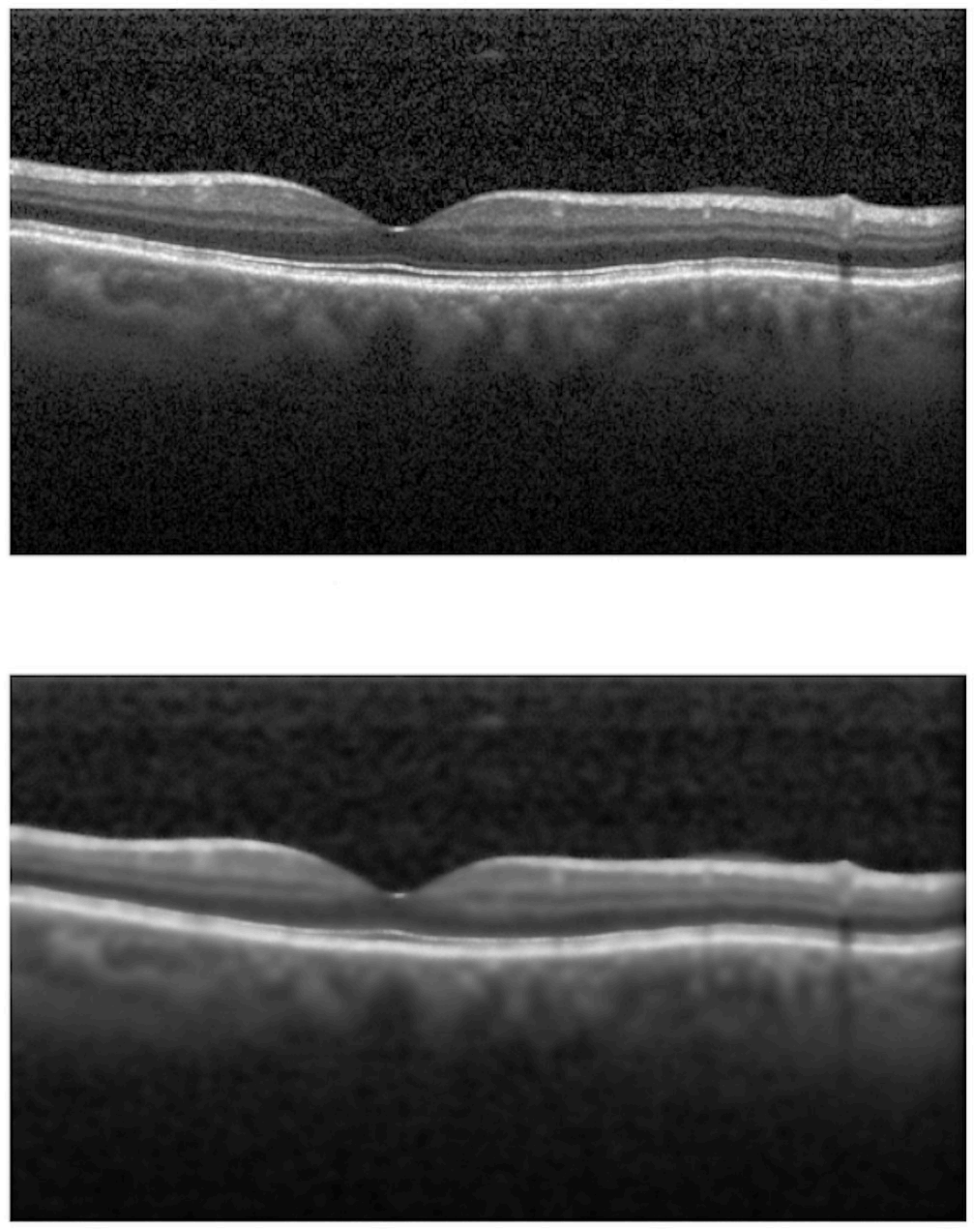
Pre-processing step. (a) No pre-processing. (b) Bilateral Filter.

The bilateral filter parameters used are: *d*= 15; *σ*_*c*_ = 100, *σ*_*s*_ = 100. We choose these parameters based on tests performed during the experiments. We chose the parameter from a predefined interval of values (d in [3, 18], *σ*_*c*_ and *σ*_*s*_ in [60, 100]) and applied it to the next step for validation, choosing the value that leads to better results. Fig 2 presents an example of the result of applying this filter.

### 2.3 Definition of the region of interest

This step has the objective of delimiting the image of each slice of the volume (B-scan) in which region the processing will be applied to identify the edges. This avoids unnecessary processing of regions that are very distant from the retina that represents the “background” of the image.

**Fig 3:**
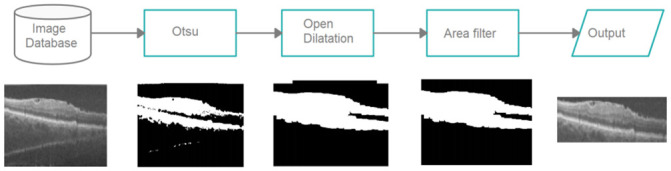
The process for defining the area of interest.

The process for defining the region of interest is done in three stages. The first one consists of applying the segmentation method of Otsu [20] to obtain an initial region. The second stage consists of the application of the morphological operations of opening (with 5 × 5 element and two iterations) and of expansion (with 5 × 5 element and seven iterations). Finally, to reach the final region, we use an elimination filter by area, where we exclude very small objects (1000 pixels). Fig 3 shows the final result of the process.

### 2.4 Initial segmentation

This step aims to further reduce the search region for the next step and separate areas of the ILM layer from the region of the RPE and BM layers. For this, we use U-Net [21], a fully connected deep neural network architecture, developed for the segmentation of medical images.

The network architecture has four downsampling layers with an activation function of the average type, where the average intensity of the window pixels is maintained. The transposed convolution is used for the enlargement stage. The convolution layers are followed by a batch normalization layer and a dropout layer, with a 20% chance of neuron deactivation. Finally, the segmentation is generated through a convolution (1 × 1), with a softmax activation function. The network is trained with the Dice Loss (S) loss function, as shown in Eq 1.

$$\begin{array}{c} S=1-(\frac{2VP}{2VP+FP+FN}) \end{array}$$ (1)

**Fig 4:**
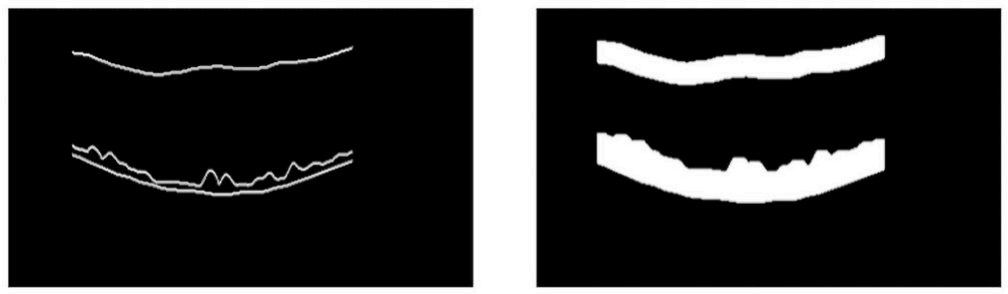
U-Net sample image. (a) Result of the ROI definition stage. (b) Image after dilation.

The U-Net training is carried out with B-scan images resulting from the stage of defining the region of interest. The images have the ground truth for the ILM, RPE, and BM borders. Morphological dilation operations are applied to the ground truth images (Fig 4), so there are two larger areas for detection. Such operations were applied since the markings are very thin, and, in the process of downsampling, the network tends to lose such information. Therefore, the dilation process tries to increase the size of the ground truth’s markings, causing it to better maintain itself during the downsampling phases. The labels inserted to the network are the borders (ILM, RPE, and BM) and bottom. For the network’s input, we resized the images to a resolution of 128 × 128. To reach the image size used, we previously tested with various sizes (64 × 64, 128 × 128, 256 × 256, 512 × 512). We verified that the size used does not significantly impact the results. This happens because this step aims to reduce the search area for the next one, performing a rough segmentation. What is sought is that the resulting area contains the layers intended to be segmented in the final step. The resize is done using the smallest image size capable of generating a rough segmentation useful for the next step, which does not create any performance loss in the segmentation process. The computational effort aspect also influenced the smallest image size. With the reduced size, we were able to train the network with more images and thus have a greater diversity of cases in the set.

**Fig 5:**
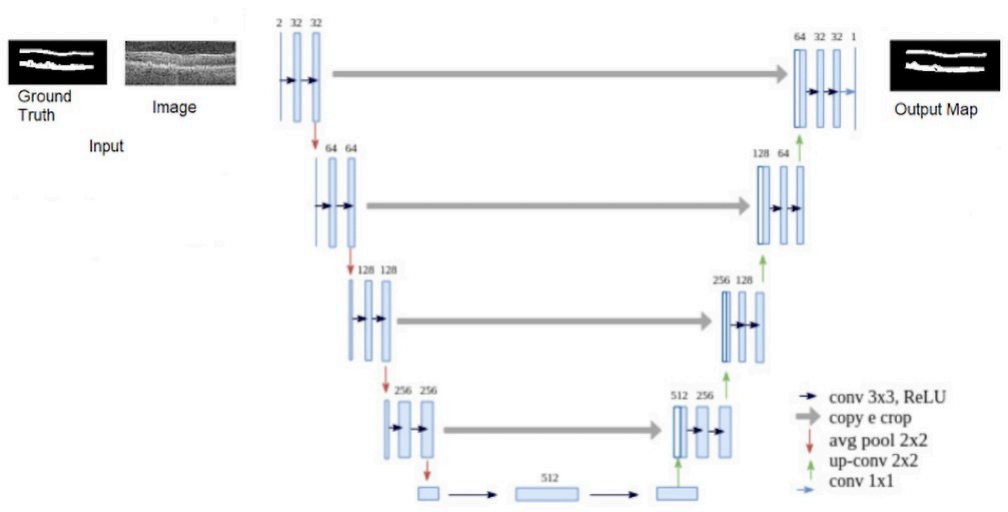
The used U-Net architecture.

The training process is shown in Fig 5, in the form of flow, indicating the order of the steps. After the images are processed, they are submitted to U-Net for training, that is, the adjustment of the weights, so the network can correctly segment the pixels corresponding to the edges.

### 2.5 Final segmentation

The DexiNed neural network was used in this work to generate the final segmentation of the edges of the layers which are the objective of the method. The architecture used was the same proposed in [22].

DexiNed is a deep neural network architecture proposed for edge detection. It consists of a stack of filters that receive an image and provide a border map. DexiNed can be seen as two subnets: a Dense extreme inception network (Dexi) and the upsampling block (UB). While Dexi is fed with the image, UB is fed with resource maps for each Dexi block. The resulting network (DexiNed) generates thin edge maps, avoiding lost edges in deeper layers [22].

**Fig 6:**
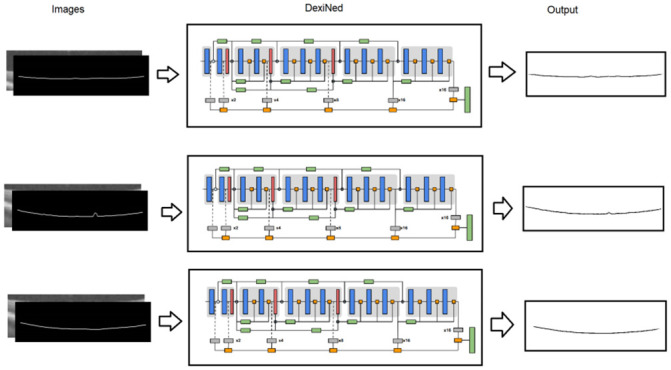
DexiNed network architecture. Font: Adapted from [22].

Fig 6 shows the structure of the network, which is composed of an encoder with 6 main blocks. The network outputs present maps in each of the main blocks to produce intermediate edge maps using an upsampling block. All edge maps resulting from the sampling blocks are concatenated to feed the stack of filters learned at the end of the network and produce a fused edge map. All six upsampling blocks do not share weights.

The blue blocks consist of a stack of two convolutional layers with a core size of 3 × 3, followed by batch normalization and ReLU as an activation function (only the last convolutions in the last sub-blocks do not possess this activation). Max pooling is used with a kernel size of 3 × 3 and stride of 2. As the architecture follows the learning process at various scales, an upsampling process is added (horizontal blocks in gray, Fig 6).

As many convolutions are carried out, all deep blocks lose important edge resources, and only one main connection is not enough, as, from the fourth convolutional layer, the loss of edge resources is more chaotic. Therefore, since block 3, the output of each sub-block is the result of the average with the border connection (orange squares in Fig 6). After max pooling and before adding to the main connection, the edge connector is defined as the average of each output sub-block (see rectangles in green, bottom side). The output of each of the main blocks feeds an upsampling block that produces an intermediate border map, and the merging of each intermediate map will generate the final result [22].

As an input to the network, we use the images delimited by the result of U-Net, thus reducing the space for searching and processing. In both training and testing, the input images were scaled from the original resolution (1000 × 512) to 992 × 128. Several tests were carried out until reaching such an image size. We realized that if the image’s width was reduced, the segmentation did not faithfully represent some curves present at the layers’ edges, especially in the RPE that presents more deformations caused by Druses and geographical atrophies. Hence, we leave the maximum width size since Dexined only works with multiples of 16. Three models were trained, one for each edge that we intend to segment (ILM, RPE, and BM).

Each model was trained with 1000 B-scans. The network parameters were the same as those used in the proposed standard architecture. A special loss function is used in this network, it was proposed by [23] and aims to adjust the network to a particular characteristic of the segmentation of edges, where 85 to 90% of the image are not edges.

### 2.6 Validation

After the segmentation of the edges of the retinal layers, it is necessary to validate the results. This work used metrics commonly used in methods that aim to achieve this objective, the mean of the Mean Absolute Error and the standard deviation (std). MAE is a performance metric that measures the accuracy of the segmentation of the retinal layer, which means absolute distances between the edge of the segmented layer and the ground truth in each column of the image. The average value and standard deviation of the T OCT scan images (size of *m* × *n*) is calculated by Eqs 2 and 3

$$\begin{array}{c} mean\left(X,Y\right)=\frac{1}{T}\sum_{t=1}^{1}(\frac{1}{n}\sum_{i=1}^{n}|X_{t}^{i}-Y_{t}^{i}|), \end{array}$$ (2)

$$\begin{array}{c} std\left(X,Y\right)=\sqrt{\frac{1}{T}\sum_{t=1}^{1}{\left(\frac{1}{n}\sum_{i=1}^{n}|X_{t}^{i}-Y_{t}^{i}|-mean\left(X,Y\right)\right)}^{2}}, \end{array}$$ (3)

where *X* and *Y* are different segmentation results, $X_{t}^{i}$ is the position on the *y* axis of A-Scan in the *t* image of OCT by the *X*, $methodY_{t}^{i}$ is the position on the *y* axis of A-Scan in the *t* OCT image by the *Y*, $X_{t}^{i}-Y_{t}^{i}$ is the absolute positioning error on the *y* axis in A-Scan *i* and B-Scan *t*.

### 2.7 Training and testing environment

All tests were implemented in the Python programming language with the help of the Keras deep learning library [24] with TensorFlow-GPU as a backend. For image processing the *Opencv* library was used [25]. For training and testing, a computer with an Intel I7 processor, 128 GB of RAM, 8 GB GeForce 1080, and Windows 10 operating system was used.

The Unet model’s training lasted approximately 360 s per epoch, and the Dexined approximately 120 s. With trained models, the system lasts about two min from the B-scan’s entry to the exit of the segmented layers with quantification metrics.

After several training sections and hyperparameters tuning, the chosen hyperparameters in the training phase were: number of epochs equal to 300, size of batch equal to 4, Adadelta optimizer with initial learning rate equal to 0.0001, decay equal to 0.095.

In the experiments, we tried to use data augmentation to verify improvements in the method’s training phase. However, increasing the sample set by applying transformations like zoom, flip, and others, did not improve layer segmentation procedure. OCT images may show a small rotation in some B-scans. There are few cases, so the magnification operations (zoom, flip, rotation, and translation) probably cannot simulate the exam.

The imaging database for patients contains only cases of intermediate AMD, thus limiting the method’s applicability to other stages of AMD.

## 3 Results

This section shows the results of experiments performed with the segmentation method of the edges of the retinal layers presented in Section 2.

The image dataset was divided into two groups. A group composed of 308 volumes of OCT was allocated to training, and another group with 76 volumes was reserved for testing. The data for each group were selected randomly, however respecting the proportion of the distribution of exams with AMD and the control group existing in the base (269: 115). Therefore, the set of images for training consisted of 220 volumes with AMD and 88 from the control group. It was used to train the networks that make up the initial and final segmentation stages. The test set was made up of 50 volumes with AMD and 26 from the control group.

For each volume of the training set, 5 B-scans were selected to train the networks, comprised of 1540 images, where 540 of which were used for validation. Since each base volume contains 100 B-scans, but not all possess a ground truth, the most central ones were chosen since it contains complete markings of the edges of the retina layers. The segmentation tests evaluated 3,600 B-scans.

**Fig 7:**
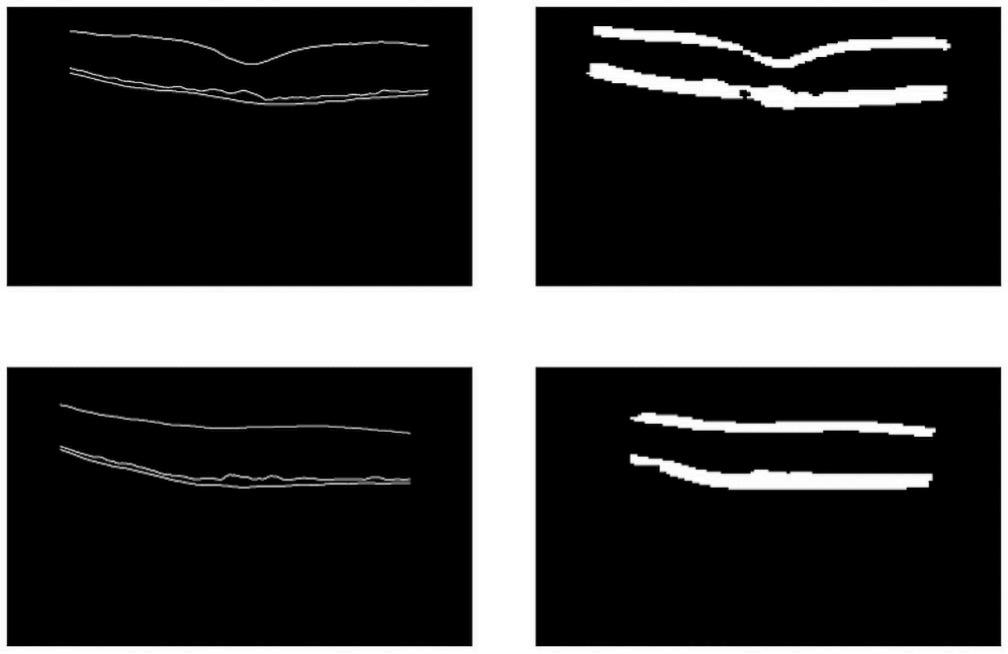
The U-Net results. On the left, the original image of the mask, and on the right, the result of the network.

The initial segmentation step has the objective of separating two areas in the image where the edges of the layers are positioned. We can verify by the structure of the retina that the ILM is separated from the other two (RPE, BM), as explained in Section 2.4. The U-net was used for this task and generated desired results, reaching a Dice of 97% in the test. In Fig 7, we can see examples of the results generated by the step.

Table 1 shows the results obtained for the segmentation of the ILM edge, the inner edge of the RPE, and the outer edge of the BM, with the results of calculating the error between our automatic segmentation method and the ground truth provided by the dataset. The values of the average of the MAE are considered low, presenting a higher value for the edges of the RPE and BM due to the fact that in retinas with AMD, drusen that cause deformations between these two layers are formed. The results showed low standard deviation values, showing that the method is very consistent, even between classes.

**Table 1:** The results of segmentation of the edges of the retinal layers.

Group	Edge	Mean Absolute Error	
		Mean	std
AMD	ILM	0.49	0.10
	RPE	0.56	0.06
	BM	0.70	0.13
Control	ILM	0.48	0.08
	RPE	0.58	0.09
	BM	0.59	0.08
All	ILM	0.49	0.09
	RPE	0.57	0.07
	BM	0.66	0.12

**Fig 8:**
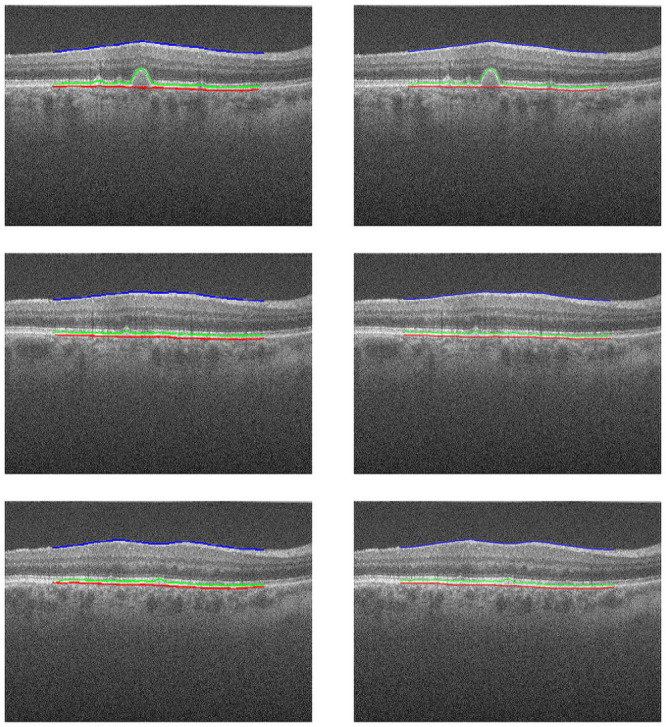
Examples of success cases. Images from the exam “AMD _1057”, in the left column are the results of our method, the dataset annotation is in the right column.

**Fig 9:**
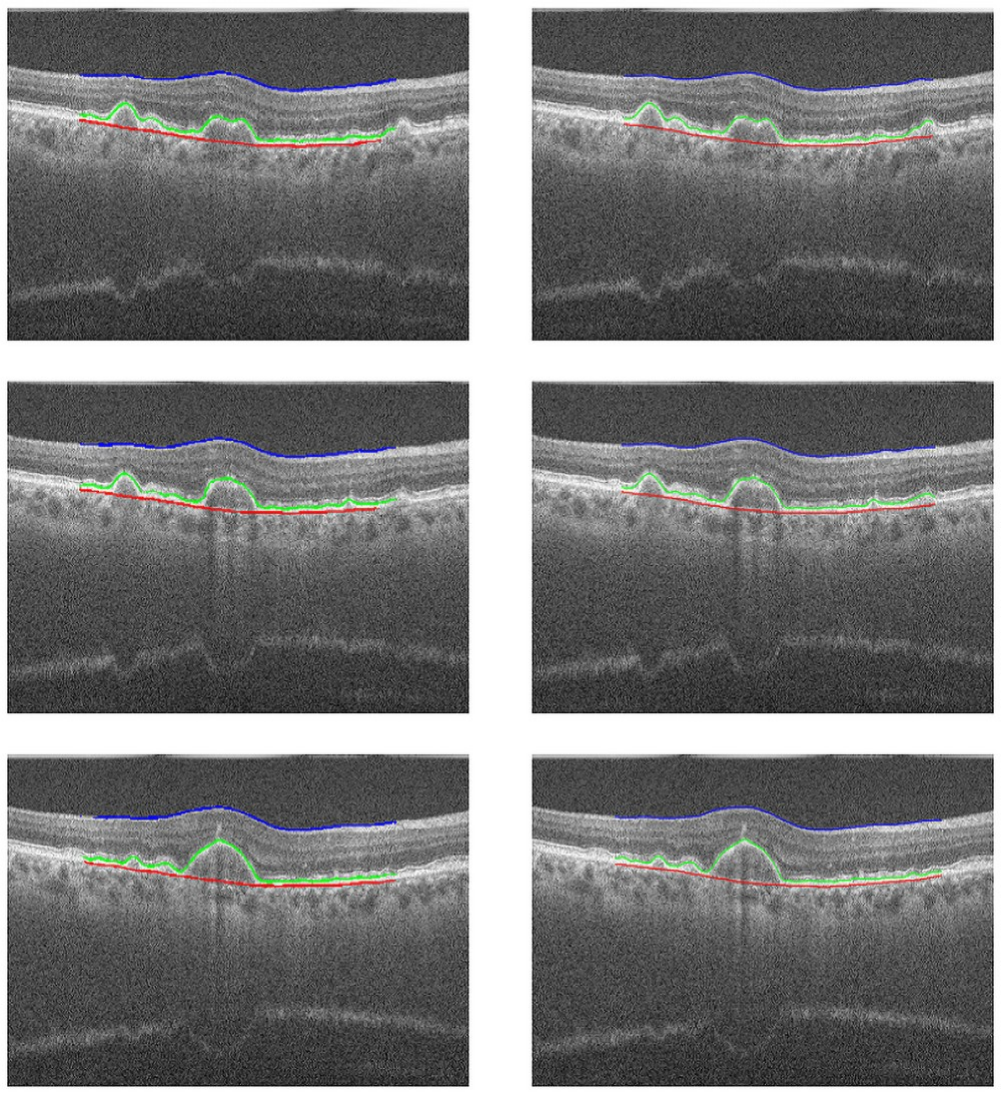
Examples of success cases. Images from the exam “AMD _1090”, in the left column are the results of our method, the dataset annotation is in the right column.

**Fig 10:**
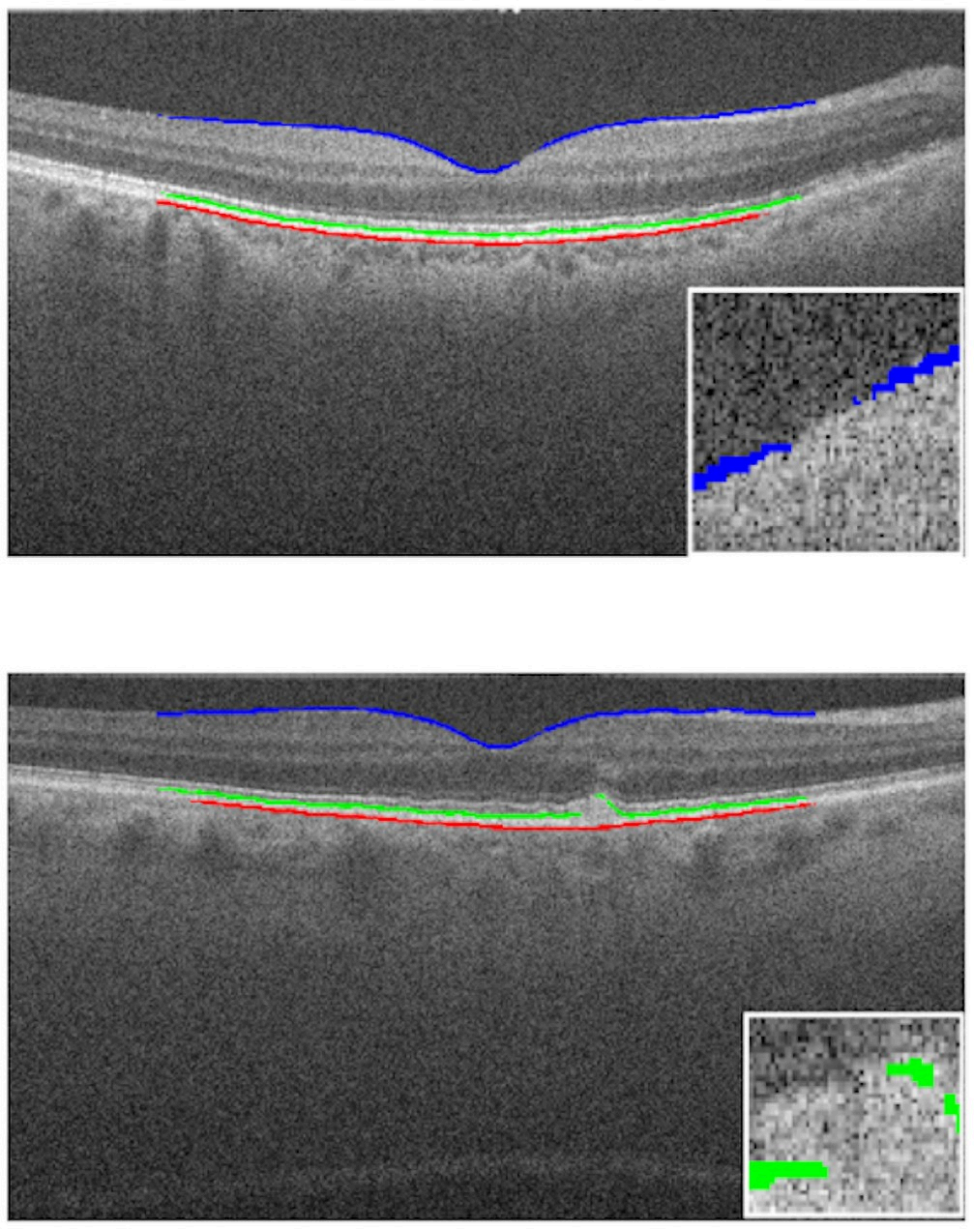
Examples of edge detection failure. (a) Exam B-scan 46 “AMD_1053”. (b) Exam B-scan 32 “AMD_1081”.

In Figs 8 and 9, we can see cases where the method managed to generate a result very close to the ground truth, a feature that stands out is the ability to detect the curvatures present at the edges of the layers, especially in the RPE and BM, as they suffer from deformations caused by drusen. In Fig 10, we have cases of error, where the method generated some flaws in the segmentation of the edges. In these errors, the method did not classify these pixels as belonging to the class of that edge, this happens when the noise level in that area makes analysis difficult, and the noise removal filter is not always able to remove and at the same time maintain the characteristics of the edges.

## 4 Discussion

The proposed method showed great robustness in the task of segmenting the edges of the retinal layers in OCT images.

It is a fully automated method. The difficulty of this task is widely recognized. The majority of studies obtained inferior results using the same dataset.

The use of a public dataset is another important feature of this work, as it allows other authors to repeat the experiments and establish comparative analysis. In this work, we use the data set from [16], which is a dataset of OCT exams with patients with intermediate AMD and healthy manifestations. It is worth mentioning that the results obtained in this experiment are very promising, and they stood out among other works proposed for the same task. In the proposed method, segmentation of the edges of the layers is a fully automated process performed entirely by two CNN models, which are very robust tools that perform implicit extraction and selection of features. This is a positive aspect because it eliminates the need to empirically determine the set of characteristics to be used in the learning process and techniques to be used in the selection of resources.

The final segmentation step is performed by DexiNed, which is a convolutional neural network created specifically for edge detection. Finally, the combination of all the techniques in this work provided a better segmentation as the results presented in Table 1 showed.

Despite presenting several positive factors, the proposed method also has some limitations.

The dataset is a very complex set, and the images possess a lot of noise, the pre-processing step that uses the bilateral filter seeks to mitigate this, but it still causes the segmentation process to be very difficult.

The image base for patients with AMD only contains intermediate cases, thus limiting the method’s performance.

Deep learning models, such as the CNNs used in this work, usually requires a large number of parameters. This set of parameters is often empirically determined by the user, which may not provide optimal results. This specific situation is another limitation of the proposed method.

All the comments mentioned have added value to this work. The many positive aspects listed in this section have allowed the proposed method to achieve good results. We emphasize that, despite some limitations, the proposed method was able to achieve significant results, and, for these reasons, we believe that the proposal provides an important contribution, as it is an automatic, innovative, robust, and promising segmentation method.

### 4.1 Comparison with related works

Table 2 presents a comparison of the proposed method with other works that used the same image dataset. The results are promising and demonstrate that the proposed method allows us to perform the segmentation of the edges with good precision. Our methodology was able to achieve values of MAE lower or very close to all the results of the related works, obtaining a standard deviation much smaller than most, showing the robustness and capability of generalization of the method. For BM and RPE, our method has the lowest values of mean absolute error and standard deviation. For ILM, the works of [14, 17] have slightly better MAE values. However, the variation is greater, reaching maximum values of 0.59 and 1.3 respectively, they also used a very small amount of images to test the methods proposed in their work, using 200 and 300 slices respectively.

**Table 2:** Comparison with related works.

Author	Method	Edge	Mean Absolute Error	
			Mean	Std
[14]	RNN-GS	ILM	0.38	0.92
		RPE	1.05	2.91
		BM	2.07	4.31
[14]	CNN-GS	ILM	1.10	7.21
		RPE	1.17	3.15
		BM	2.31	4.60
[14]	FCN-GS	ILM	0.65	4.24
		RPE	1.03	2.97
		BM	1.53	3.50
[15]	CapsNet	ILM	0.75	3.42
		RPE	0.93	0.86
		BM	1.09	2.49
[17]	DeepForest	ILM	0.47	0.12
		RPE	0.99	0.48
		BM	1.24	0.52
[18]	WAVE	ILM	0.68	4.09
		RPE	1.22	2.80
		BM	1.90	3.01
proposed method	DexiNed	ILM	0.49	0.09
		RPE	0.57	0.07
		BM	0.66	0.12

## 5 Conclusion

Early diagnosis of age-related macular degeneration is very important as it is an eye disease that can cause visual impairment and blindness. One of the main characteristics of the disease is the formation of druses, which cause deformations in the retina between the layers of the retinal pigment epithelium and the Bruch’s membrane. In this article, we proposed a methodology for segmenting the edges of three layers of the human retina; lower limiting membrane, retinal pigment epithelium, and Bruch’s membrane, which are important for the diagnosis of AMD.

The proposed method has four steps, pre-processing with a bilateral filter that eliminates noise and highlights the edges, two steps to define the area of interest, and a final segmentation step with DexiNed to find each layer’s limits. The experiment results show that our method is robust for healthy retinas and age-related macular degeneration. The statistics for mean absolute errors and standard deviation are better than those found in state of the art for the layers of the retinal pigment epithelium and Bruch membrane. Our method differs from the literature in that it presents a model specialized in segmenting each of the edges, ILM, RPE, and BM.

Despite having obtained promising results, the proposed method can be improved in some aspects. To overcome some of the current limitations of our method, we present the following research directions.

Search for other databases with severe dry AMD and wet AMD cases to test the method in such conditions.

One of the aspects of research that can contribute to the improvement of the proposed method is the investigation of other pre-processing techniques for removing adaptive noise such as the Wiener filter since the excess noise and variation between images is a large issue.

The visualization of the edges of the ILM, RPE, and BM layers is also very important for the diagnosis of another disease, diabetic retinopathy, so this method can be incorporated into a method for the diagnosis of this pathology from images of exams of patients affected by this illness.

Finally, as the models of deep neural networks generally have a large number of empirically determined parameters, we suggest the use of hyperparameter definition and optimization strategies to obtain more tuned architectures for both models.
